# Pancreatic insulinoma co-existing with gastric GIST in the absence of neurofibromatosis-1

**DOI:** 10.1186/1477-7819-7-18

**Published:** 2009-02-13

**Authors:** Edward Alabraba, Simon Bramhall, Brendan O'Sullivan, Brinder Mahon, Philippe Taniere

**Affiliations:** 1 University of Birmingham and UHB Foundation NHS Trust, Birmingham, UK; 2 UHB Foundation NHS Trust, Birmingham, UK

## Abstract

**Background:**

Gastrointestinal stromal tumours (GIST) frequently occur in patients with neurofibromatosis type 1 (NF-1). It has been reported that GIST may co-exist with pancreatic endocrine tumors but this has only been in association with NF-1.

**Case presentation:**

A 76 year old woman presented with a 12 month history of hypoglycaemia symptoms. Abdominal CT scan demonstrated a 13 mm insulinoma localized in the tail of her pancreas. She was commenced on diazoxide and later underwent surgery for enucleation of insulinoma when a small (< 1 cm) incidental tumour was discovered on her stomach wall which was identified as GIST.

**Conclusion:**

This is the first case report of a pancreatic insulinoma co-existing with a GIST in a patient without NF-1. In addition, we make the first report of rapidly growing cystic GIST recurrence following resection of a primary GIST tumour.

## Background

Gastro-entero-pancreatic (GEP NE) tumours have an incidence of around 30/million/population/year [1]. Pancreatic islet cell tumours including insulinomas represent the 2nd most common type of GEP NE tumour after carcinoid tumour [2].

GIST most commonly occur sporadically, but show increased tendency in patients with NF1 [3,4]. A small number of pancreatic neuroendocrine tumors have been described in NF-1 patients [5,6]. There have been only nine reports of GIST associated with GEP NE tumours but these have all been diagnosed in patients with NF-1 [7-14] (Table 1).

**Table 1:** Co-existent GIST and NET in patients with NF-1

**GIST**	**Neuroendocrine tumor**	**Reference**
Small bowel	Papilla of Vater	[10]

Small bowel	Pheochromocytoma	[9]

Small bowel	Papilla of Vater; domatostatinoma	[12]

Small bowel	Pancreatic head gastrinoma	[14]

Small bowel	Duodenum neuroendocrine carcinoma	[7]

Small bowel	Pheochromocytoma	[11]

Small bowel	Pheochromocytoma	[8]

Small bowel	Pheochromocytoma	[10]

Large bowel	Pheochromocytoma	[10]

Here we report the first case of insulinoma associated with a GIST in a patient not diagnosed with NF-1 as confirmed by the pre-operative endocrine assessment.

## Case presentation

A 76 year old woman presented with a 12 month history of hypoglycaemia symptoms. The patient underwent a series of tests including measurement of overnight fasting plasma glucose, measurement of plasma levels of glucose; insulin; and c-peptide during a hypoglycaemic episode, measurement of urinary sulphonylurea, and radiological imaging with a CT scan. Abdominal CT scan demonstrated a 13 mm insulinoma localized in the tail of her pancreas. She was commenced on diazoxide and later underwent surgery for enucleation of insulinoma when a small (< 1 cm) incidental tumour was discovered on her stomach wall. The small incidental tumour was an exophytic gastric wall lesion that had not been detected pre-operatively and revealed no suspicious features suggestive of invasion or spread on intra-operative inspection. For this reason the lesion was excised with diathermy off the surface of the gastric wall, the defect being closed with Polydioxanone suture, and no frozen sections were taken to assess invasion/spread. Macroscopically the lesion was completely excised. The nature of the lesion was unknown at this time. Recovery was uncomplicated and she was discharged home a week later.

**Figure 1:**
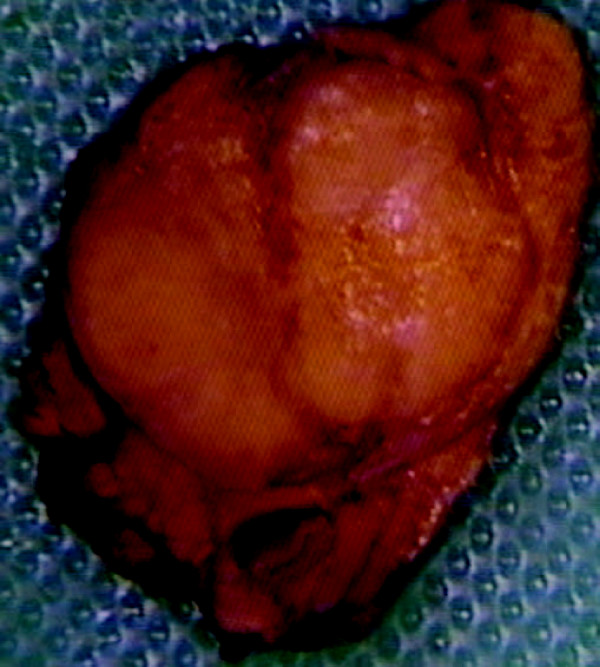
**Gross histological appearance of insulinoma showing typical red-brown appearance of tumour**.

The pancreatic lesion (figure 1) was composed of homogeneous soft tissue that had the typical red brown colour of such lesions although paler than usually seen, measured 13 mm in diameter, and its histology showed a well differentiated neuroendocrine tumour with nesting and trabecular pattern. The tumour cells were regular, their nuclei were round with vesicular chromatin and no mitosis was identified. Numerous psammoma bodies were seen in tumour nests and there was abundant hyalinised stromal reaction with no remarkable inflammation or necrosis. Immunohistochemistry showed strong positivity with chromogranin A, synaptophysin, progesterone, oestrogen-β and insulin markers. Very occasional cells were stained by somatostatin antibodies whilst pancreatic polypeptide, gastrin, amyloid and CD117 (C-kit) immunostaining were negative. There was a low proliferation index with less than 1% of tumour cells being stained by Ki67 antibody. Also, no vascular invasion was noted. The tumour was encapsulated and looked completely excised. The insulinoma was classified as T1NxMx.

**Figure 2:**
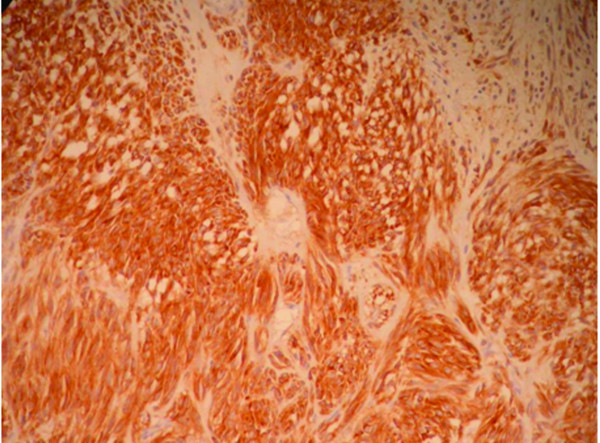
**Gastric wall GIST tumour cells were strongly positive for CD117 (c-kit) marker (×100)**.

The gastric lesion was a 6 mm firm whitish nodule in the upper wall of her stomach and its histology revealed an epithelioid cell tumour. Mitotic rate was very low and no necrosis was noted. Immunohistochemistry showed uniform positivity with C-kit (figure 2) and CD34 markers. Desmin, smooth muscle actin, S100, cytokeratin and neuroendocrine markers were all negative. Ki67 immunostaining revealed very low proliferation index. We attempted performing mutation analysis on the GIST tumour but the DNA obtained was not of sufficiently good quality for the assay. We were unable to comment on the nature of the tumour margin because the GIST lesion was excised without any adjacent gastric wall tissue. The overall appearance was that of an epithelioid kit positive incidental GIST tumour and the lesion was incompletely excised.

**Figure 3:**
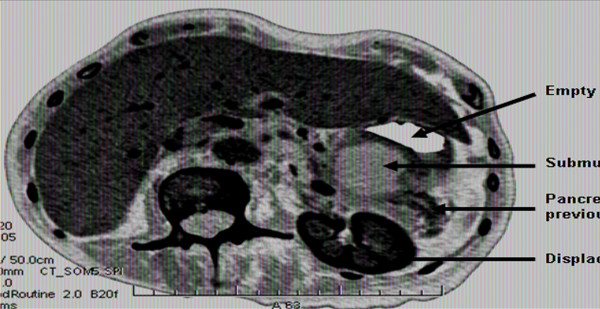
**CT abdomen showing cystic recurrent GIST located in the region of the pancreatic tail**.

The patient was re-admitted 6 months later with weight loss, anorexia and vague symptoms. She had a CT scan which showed a 3.9 × 3.9 cm fluid attenuation mass superior to the tail of her pancreas (figure 3) displacing the stomach anteriorly. Endoscopic ultrasound confirmed a solid/cystic irregular mass involving the stomach wall, and in close relation to the pancreatic tail.

**Figure 4:**
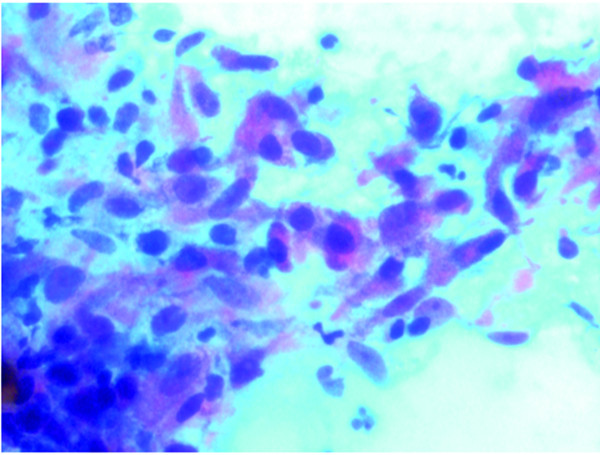
**EUS-guided aspiration biopsy cytology of recurrent GIST showing clusters of atypical epithelioid and spindle cells (PAP ×400)**.

She subsequently underwent oesophagogastroduodenoscopy-guided endoscopic ultrasound confirming an ill-defined mass extending into the stomach wall. Cytology revealed groups of spindly and epithelioid cells highly suggestive of recurrent/metastatic GIST but not indicative of a neuroendocrine tumour (figure 4). She was managed symptomatically, discharged and had another abdominal CT scan 7 months later that showed that the lesion had not significantly grown in size, but did appear to have formed 2 well defined cystic components measuring 29 × 28 mm, and 21 × 24 mm. Owing to the small size of the primary GIST and the frail state of the patient, she was not treated with Imatinib (Glivec) or offered further surgery but has subsequently been regularly reviewed by CT scan surveillance of the lesion and has fortunately had no evidence of tumour size increase or metastases.

## Discussion

The first important message in our case is that a GIST can occur simultaneously with an insulinoma in the absence of NF-1. The GIST tumour was only incidentally discovered by the operating surgeon during enucleation of the insulinoma in our case. The literature contains only 9 published reports of the simultaneous occurrence of GIST and GEP NE tumour in NF-1 patients [7-12] (Table 1). None of the reported cases of co-existent GIST and GEP NE tumours in NF-1 patients has ever involved an insulinoma and in fact the only report of hypoglycaemia associated with GIST was attributed to a rare paraneoplastic syndrome caused by over-expression of insulin-like growth factor II [15] in an advanced gastric GIST. It is debatable whether or not incomplete excision of the gastric GIST could have been avoided but because the gastric GIST was only discovered incidentally, showed no suspicious features on gross inspection, and the opinion of another experienced surgeon sought during the surgical procedure was in concordance with that of the operating surgeon, it was decided to proceed with simple excision of the gastric lesion with minimal dissection as was done.

The other unique message in our case is the observed rapid recurrence of the GIST following the resection of the primary gastric wall GIST. Small GISTs, like the initial one in our case, are usually an incidental surgical finding, while large tumours are usually symptomatic. In our case, following resection of the insulinoma and the small incidental gastric GIST, the GIST recurred locally with rapid enlargement. The tumour showed marked cystic transformation probably as result of central necrosis and liquefaction. The reason for the rapid recurrence of the GIST is unclear but evidence from animal studies demonstrates that a primary tumour can inhibit its remote metastases via circulating angiostatin such that after primary tumour removal, the inhibition is removed and metastases become vascularized and grow rapidly [16]. We suspect that in our case, the primary GIST may have had a suppressive effect on satellite micro-lesions which was lost when the GIST was resected leading to aggressive local recurrence. An additional finding is the unique cystic change in the recurrent GIST. Although it has been reported that a rapidly growing primary GIST can form a lobulated cystic mass lesion [17], such rapid cystic mass recurrence has never been reported in the literature following resection of a small incidental tumour. We were surprised by the recurrence of the GIST lesion as the initial GIST of the gastric wall had a low mitotic rate and also a low ki-67 index. We accept that the mechanism of the GIST 'recurrence' may be one of four possibilities: true recurrence of the primary gastric wall GIST, undetected primary at initial surgery when the stomach wall GIST was removed, residual tumour from initial surgery when the stomach wall GIST was removed, or metastatic disease related to the primary gastric wall GIST.

In summary, this is the first case report of a non-NF-1 patient that had a pancreatic insulinoma co-existent with a GIST, and also, the first case report of a GIST that underwent rapid cystic recurrence following resection of the primary GIST lesion. Unfortunately our unique case report raises more questions than provides answers because there are very few cases of NF-1-related concurrent GEP NE tumours and GISTs and also very little understanding of the prognostic implications of such concurrent tumours. Available evidence suggests that mutations in the NF-1 gene might be involved in the pathogenesis of GIST in NF-1 patients [18]. However it is unknown if the same mutation exists in NF-1 patients with co-existent GISTs and GEP NE tumours, and more importantly, it is unknown if there are such mutations in non-NF-1 patients with co-existent GISTs and GEP NE tumours. Our unit routinely performs gene mutation analysis on GIST tumours but unfortunately there is a 2% failure rate in our mutation analysis and attempts to analyse the primary GIST lesion in this case were unsuccessful. Completing this mutation analysis would have greatly enhanced our understanding of the unusual co-existence of this tumour in the setting of a non-NF-1 patient, and, also shed more light on its unusual pattern of recurrence.

## Consent

Written informed consent was obtained from the patient for publication of this case report and any accompanying images. A copy of the written consent is available for review by the Editor-in-Chief of this journal.

## Competing interests

The authors declare that they have no competing interests.

## Authors' contributions

EA conceived and designed the report, analyzed all the reports and drafted the manuscript. SB performed surgery on the patient and participated in designing the report. BO performed mutation analysis on the GIST tumour and participated in the analysis of histological specimens. BM performed the radiological procedures on the patient and participated in designing the report. PT analyzed and reported on all the histological specimens in the case and participated in designing the report. All authors read and approved the final manuscript.
